# Gratuitous Services to Clergymen

**Published:** 1880-05

**Authors:** 


### GRATUITOUS SERVICES TO CLERGYMEN.

IN regard to charging the clergy for medical services, the following resolution was adopted at the meeting of the Allegan County, Mich., Medical Association, July 26, 1877:

"*Resolved,* That the custom of giving our professional services to clergymen and their families gratuitously is unjust to a large class of our patrons, whose incomes are much less than the average incomes of theirs, and that we will hereafter adopt the practice of charging them the same as others."—*Med. Record.*

